# LGBTQ+ Identity Social Support and Care Access among LGBTQ+ Caregivers of Individuals Living with Mild Cognitive Impairment, Alzheimer's Disease, and Related Dementias

**DOI:** 10.1002/alz70858_097417

**Published:** 2025-12-24

**Authors:** Nik M. Lampe, Ellesse‐Roselee L. Akré, Harry Barbee, Tara McKay

**Affiliations:** ^1^ University of South Florida, Tampa, FL, USA; ^2^ Johns Hopkins University Bloomberg School of Public Health, Baltimore, MD, USA; ^3^ Vanderbilt University, Nashville, TN, USA

## Abstract

**Background:**

This study examines associations of caregiver status with LGBTQ+‐identity social support, relationships, and care outcomes among LGBTQ+ older adults.

**Method:**

We use LGBTQ+ Social Networks, Aging, and Policy Study Wave 3 data (QSNAPS; *N* = 982). Descriptive and logistic regression analyses were conducted to examine associations between caregiver status and outcomes concerning LGBTQ‐identity social support, care, and care recipient relationships.

**Result:**

Eighty respondents (8.1%) care for 90 individuals with neurocognitive disorders, a majority of whom are parents of the respondent. Caregivers were half as likely to have family support (*p* <0.05); 40.6% less likely to have coworker support (*p* <0.1); and 45.6% less likely to have neighbor support (*p* <0.05) for LGBTQ+ identities. Caregivers were more likely to receive practical help from others (*p* <0.01) but experienced concerning issues related to their own access to healthcare.

**Conclusion:**

Understanding LGBTQ+‐identity social support and care access can inform targeted interventions to reduce LGBTQ+ caregiver health disparities.

